# 1-Palmitoyl-2-linoleoyl-3-acetyl-rac-glycerol (PLAG) attenuates gemcitabine-induced neutrophil extravasation

**DOI:** 10.1186/s13578-018-0266-7

**Published:** 2019-01-03

**Authors:** Jinseon Jeong, Yong-Jae Kim, Do Young Lee, Byoung-Gon Moon, Ki-Young Sohn, Sun Young Yoon, Jae Wha Kim

**Affiliations:** 1 Cell Factory Research Center, Division of Systems Biology and Bioengineering, Korea Research Institute of Bioscience and Biotechnology, 125 Gwahak-ro, Yuseong-gu, Daejeon, 305-333 Republic of Korea; 2 Department of Functional Genomics, University of Science & Technology, Daejeon, Republic of Korea; 3 Division of Global New Drug Development, ENZYCHEM Lifesciences, Jecheon, 27159 Republic of Korea

**Keywords:** PLAG, Gemcitabine, Chemotherapy induced neutropenia (CIN), Chemokine, Reactive oxygen species, NADPH oxidase 2

## Abstract

Cancer patients treated with chemotherapy often experience a rapid decline of blood neutrophils, a dose-limiting side effect called chemotherapy-induced neutropenia. This complication brings about dose reductions or cessation of chemotherapy during treatment of cancer patients because a rapid decline of neutrophil counts increases susceptibility to infection. Here, we found that 1-palmitoyl-2-linoleoyl-3-acetyl-rac-glycerol (PLAG) attenuates gemcitabine-induced neutrophil extravasation via the inhibition of neutrophil-attracting chemokine production in macrophages using in vivo and in vitro approaches. A single intraperitoneal administration of gemcitabine induced the migration of circulating neutrophils into the peritoneal cavity in normal mice, and PLAG effectively decreased neutrophil migration by inhibiting the expression of adhesion molecules, L-selectin and LFA-1. Inhibition of CXCR2 by its antagonist, reparixin, abrogated gemcitabine-induced neutrophil migration, indicating that chemokines produced by gemcitabine mainly support neutrophil activation. In vitro experiments demonstrated that PLAG inhibited NADPH oxidase 2 (NOX2)-mediated reactive oxygen species production induced by gemcitabine, which is the upstream of MIP-2 and/or CXCL8. Importantly, PLAG down-regulated gemcitabine-induced membrane translocation of the cytosolic NOX subunit, Rac1, and phosphorylation of p47phox. The activation of upstream signaling molecules of p47phox phosphorylation, phospholipase C β3 and protein kinase C, were effectively regulated by PLAG. We also demonstrated that 1-palmitoyl-2-linoleic-3-hydroxyl-rac-glycerol (PLH), the natural form of diacylglycerol, has no effects on gemcitabine-induced CXCL8 production and dHL-60 migration, suggesting that an acetyl group at the third position of the glycerol backbone may have a key role in the regulation of neutrophil activation. Altogether, this study suggests the potential of PLAG as a therapeutic strategy to modulate chemotherapy-induced neutrophil activation for cancer patients undergoing chemotherapeutic treatment.

**Electronic supplementary material:**

The online version of this article (10.1186/s13578-018-0266-7) contains supplementary material, which is available to authorized users.

## Introduction

Neutrophils are the most abundant white blood cell in circulation and play a key role in the first-line of defense against foreign substances and/or invading pathogens [1, 2]. Cancer patients undergoing treatment with chemotherapeutics often suffer from a rapid decline of circulating neutrophils [3]. This complication, which is called chemotherapy-induced neutropenia (CIN), increases the patient’s susceptibility to infection, which necessitates dose reduction or cessation of chemotherapy [4]. Unfortunately, exact mechanism**s** of CIN remains unknown, for that reason, current treatment options rely mostly on promoting production of granulocytes and precursor cells in the bone marrow. Filgrastim, the recombinant human granulocyte-colony stimulating factor (rhG-CSF), is one of the few treatment options since its natural form is a key regulator of neutrophil differentiation and proliferation [5–7]. Therefore, a better understanding of how neutropenia arises in response to chemotherapy would be the key for the development of new pharmaceuticals.

The immunosuppressive effect of chemotherapeutics ha**s** long been considered as an immediate cause of neutrophil depletion due to its high susceptibility to cytotoxic activity [8, 9]. However, increasing evidences suggest that the immune systems are activated by many chemotherapeutics [10]. For example, paclitaxel activates mouse macrophages and dendritic cells (DCs) by binding to mouse toll like receptor 4 (TLR4) and mimicking bacterial lipopolysaccharides (LPS) [11]. In animal models with chemotherapy-induced peripheral neuropathy (CIPN), there are several reports of increased neutrophil activity and infiltration in the peripheral nervous system (PNS) and dorsal root ganglion (DRG) following injection with anti-cancer agents [12, 13].

Gemcitabine is a nucleoside deoxycytidine analogue, which is a commonly used anti-cancer agent with a high incidence of CIN [14]. It is an antimetabolite that induces cell death by interfering with enzymes that are necessary for nucleic acid production during S phase of the cell cycle [15, 16]. Gemcitabine also acts by generating reactive oxygen species (ROS) through upregulation of NADPH oxidase (NOX) activity [17]. In the previous study, gemcitabine enhanced pancreatic cancer stemness through NOX-mediated ROS production [18]. Gemcitabine also enhances the production of CXC motif chemokine ligand 8 (CXCL8) and/or macrophage inflammatory protein 2 (MIP-2; a murine homologue of CXCL8), which are the primary chemokines that recruit neutrophils to damaged tissue at early phases [19, 20]. However, a direct association between NOX-mediated ROS and the chemokine production in gemcitabine—treated condition remains unclear.

1-Palmitoryl-2-linoleoyl-3-acetyl-rac-glycerol (PLAG) was originally isolated from the antlers of sika deer and chemically synthesized as a single compound that is identical to the natural form [21]. A previous study showed that PLAG exerts a synergistic therapeutic effect with long acting G-CSF (pegfilgrastim) to treat CIN by modulating neutrophil migration [22]. Also, PLAG reduced the infiltration of neutrophils into the arthritic joints by inhibiting IL-6/STAT3/MIP-2 signaling cascade in rheumatoid arthritis [23]. In this study, we aimed to demonstrate low neutrophil counts in blood after gemcitabine treatment partially due to neutrophil chemotaxis from blood to tissues along a MIP-2 gradient, and investigated the protective effect of PLAG on the maintenance of circulating neutrophil counts using a murine model of gemcitabine-induced neutropenia. We further demonstrated the molecular mechanism in which gemcitabine promotes CIN by inducing the chemokine gradient through the activation of PLCβ3/PKC/NOX2/ROS/MAPK signaling cascade. Together, we show that PLAG has a therapeutic effect against CIN via modulating NOX2-mediated ROS generation and downstream ROS signaling pathways, which leads to chemokine production.

## Materials and methods

### Chemicals

PLAG and PLH were obtained from Enzychem Lifesciences Corporation (Daejeon, South Korea). Gemcitabine hydrochloride was purchased from Dong-A ST (Seoul, South Korea). Reparixin was purchased from MedChem Express (NJ, USA). *N*-acetyl-l-cycteine, diphenyleneiodonium were purchased from Sigma-Aldrich (MO, USA). U73122 and Rottlerin were purchased from ENZO Life Sciences (NY, USA).

### Animals

Male BALB/c mice (6–8 weeks of age, 20–22 g) were purchased from Koatech Corporation (Pyeongtaek, South Korea), and male BALB/c Slc-*nu* mice (5 weeks of age, 17–21 g) were purchased from Central Lab Animal Inc. (Seoul, South Korea). The mice were maintained in a specific pathogen-free facility under consistent temperature and 12-h light/dark cycles. All experimental procedures were approved by the Institutional Animal Care and Use Committee of the Korea Research Institute of Bioscience and Biotechnology (Daejeon, South Korea) and performed in compliance with the National Institutes of Health Guidelines for the care and use of laboratory animals and Korean national laws for animal welfare.

### Animal studies

For gemcitabine-induced neutropenia mice model, male BALB/c mice were intraperitoneally (i.p.) injected with 50 mg/kg gemcitabine to induce neutropenia. PLAG was diluted in phosphate buffered saline (PBS) and then orally administrated at a dose of 50 or 250 mg/kg/day. The normal control group was administered PBS only during the experiment. At 15 h after gemcitabine treatment, the whole blood was collected from the orbital sinuses using capillary tubes (Kimble Chase Life Science and Research Products LLC, FL, USA) and collection tubes containing K3E-K3EDTA (Greiner Bio-One International, Kremsmünster, Austria). To obtain peritoneal cells, 5 ml of cold PBS was injected to the left side of peritoneal wall using a 5 ml syringe, and the fluid was aspirated from the peritoneum. The collected cells were counted by complete blood count (CBC) analysis using Mindray BC-5300 auto-hematology analyzer (Shenzhen Mindray Biomedical Electronics, Guangdong Sheng, China).

For a human tumor xenograft model, athymic nude mice (n = 10 per treatment group) were inoculated subcutaneously on the right flank 10^7^ RPMI8226 cells in 0.2 ml PBS on day 0. Chemotherapy and PLAG administration were begun when tumor volume reached between 81 and 118 mm^3^ on day 12. Gemcitabine was administered (i.p.) twice weekly for 4 weeks, and PLAG was orally administered once per day for 4 weeks. On day 40, all mice were sacrificed, and tumor weights were measured.

### Flow cytometry

The collected whole blood was incubated with ACK Lysing Buffer (Life Technologies, Karlsruhe, Germany) for 5 min to remove erythrocytes, and washed with PBS containing 1% fetal bovine serum (FBS) (Welgene, Kyungsan, South Korea) three times. The cells were subsequently stained with FITC-conjugated anti-Ly6G and PE-Cy7-conjugated anti-CD11b antibodies (BD biosciences, NJ, USA) to determine the circulating neutrophil population. To investigate the expression of adhesion molecules on Ly6G+/CD11b+ cells, the cells were further stained with APC-conjugated anti-L-selectin and APC-conjugated anti-LFA-1 antibodies (BD biosciences). The stained cells were analyzed with a FACSVerse flow cytometer (BD biosciences), and the data were processed with FlowJo software (TreeStar, OR, USA).

### Cell culture

A human promyelocytic leukemia cell line, HL-60, and a human monocytic cell line, THP-1, were obtained from American Cell Culture Collection (ATCC, USA). The cells were maintained in RPMI-1640 (Welgene) supplemented with 10% heat-inactivated FBS (Welgene), 2 mM l-glutamate, 100 U/ml penicillin and 0.1 mg/ml streptomycin (Welgene) and cultured at 37 °C in a humidified atmosphere with 5% CO_2_.

For bone marrow-derived macrophages (BMDMs), the femur and tibia were collected from balb/c mice, and bone marrow cells were flushed with complete DMEM supplemented with 10% FBS and 0.1 mg/ml streptomycin. Red blood cells were removed using ACK lysing buffer (Gibco, NY, USA), and the cell suspension were filtered through a 40-μm cell strainer (Corning, NY, USA) to remove any cell clumps. The single cell suspensions were cultured for 7d in complete DMEM supplemented with 30% conditioned medium of L929 mouse fibroblasts in order to fully differentiate the cells into BMDMs. The culture medium was replaced every 2d, and more than 95% of the differentiated cells were F4/80+ when analyzed by flow cytometry (Additional file 1: Fig. S1A).

### HL-60 differentiation and migration assay

HL-60 cells were differentiated (dHL-60) to neutrophil-like cells in the culture medium with the addition of 1.3% DMSO (Sigma) for 5 days in a humidified atmosphere at 37 °C without changing the medium. The differentiated cells were stained with anti-CD11b-PE and were analyzed by flow cytometry (Additional file 1: Fig. S1B). Migration assay was performed as described previously with minor modifications [24]. Briefly, THP-1 cells were pretreated with various concentrations of PLAG for 1 h and stimulated with gemcitabine for 24 h. The THP-1 supernatant was placed on the bottom chamber as chemoattractant. The differentiated HL-60 cells (1 × 10^6^ cells/100 μL) were transferred to the upper chamber of Transwell^®^ 24-well plates with 5 μm polycarbonate membrane filters (Corning) and were incubated for overnight. The total number of migrated cells was counted by trypan blue staining using a hemocytometer.

### Enzyme-linked immunosorbent assay (ELISA)

The concentration of murine MIP-2 in serum and cell supernatants was measured using MIP-2 ELISA kit (R&D systems, MN, USA), and the level of human CXCL8 from cell supernatants was detected by human CXCL8 ELISA kit (BD biosciences) according to the manufacturer’s instructions. Optical densities were measured at 450 nm using a Bio-Rad Model 550 microplate reader (Bio-Rad Laboratories, CA, USA). The chemokine levels were calculated from a standard curve generated by a curve-fitting program.

### Reverse transcription polymerase chain reaction (RT-PCR)

Total RNAs from cells were isolated using TRIzol^®^ reagent (Invitrogen, CA, USA) according to the manufacturer’s instructions. RT-PCR was performed using PCR reagent (Bioassay, Daejeon, South Korea). Complementary DNA (cDNA) was synthesized from total RNA using a RT kit (Bioassay), followed by conventional PCR. The primers used in this study are as follows: human CXCL8, 5′-AGGGTTGCCAGATGCAATAC-3′ and 5′-GTGGATCCTGGCTAGCAGAC-3′; mouse MIP-2, 5′-AGTGAACTGCGCTGTCAATG-3′ and 5′-CTTTGGTTCTTCCGTTGAGG-3′; GAPDH, 5′-CCATCACCATCTTCCAGGAG-3′ and 5′-ACAGTCTTCTGGGTGGCAGT-3′.

### Membrane fractionation and immunoblotting

Total cells were lysed on ice for 30 min in RIPA buffer composed of 50 mM Tris-HCl (pH 7.4), 150 mM NaCl, 2 mM EDTA, 1% NP-40, 0.5% sodium deoxycholate, 0.1% SDS, 1 mM phenylmethylsulfonyl fluoride (PMSF), 10 mM sodium fluoride, 2 mM sodium pyrophosphate, 10 mM β-glycerophosphate, 10 mM sodium orthovanadate. Membrane-and-cytoplasmic protein fractions of cultured cells were obtained with Mem-PER Plus Membrane Protein Extraction Kit (Thermo Scientific™, MA, USA) according to the manufacturer’s instructions. The lysates were centrifuged at 13,000 rpm for 20 min at 4 °C and protein concentrations were determined using the Bradford assay (Bio-Rad Laboratories). Denatured samples were mixed with a 5× SDS-PAGE loading buffer and heated to 100 °C for 15 min. The samples were separated on the 10% of SDS-PAGE gel and transferred to polyvinylidene difluoride (PVDF) membranes (Merck Millipore Corporation, MA, USA). Membranes were blocked with 5% non-fat milk in PBS (10 mM Tris-HCl, pH7.5, 150 mM NaCl) for 1 h and probed with primary antibodies against ERK1/2, phospho-ERK1/2, P38, phospho-P38, SAPK/JNK, phospho-SAPK/JNK, Na,K-ATPase, α-Tubulin, phospho-PLCβ3, PLCβ3, phospho-PKCα/βII, phospho-PKCδ, PKCδ and β-actin from Cell Signaling Technology (MA, USA), Rac1 from Merck Millipore Corporation), p47phox and phospho-p47phox from Invitrogen™ for overnight at 4 °C. The blots were washed and incubated with appropriate secondary antibodies and visualized using Pierce™ ECL Western Blotting Substrate (Thermo Scientific™).

### Intracellular ROS measurement

A total of 1 × 10^6^ BMDMs and THP-1 were seeded, cultured, and subsequently exposed to various concentrations of PLAG with gemcitabine (10 μg/mL) for 3 h. The cells were then incubated with the ROS-sensitive probe CM-H_2_DCFDA (Invitrogen™) for 30 min at 37 °C in the dark. After incubation, the cells were washed 3 times with PBS and immediately analyzed using FACS verse (BD biosciences) with an excitation/emission peak at 495/527 nm. A total of 10,000 cells were counted in each determination, and results presented are mean ± S.E. of three independent experiments. Intracellular ROS production was also measured with a confocal laser scanning microscope (Zeiss LSM 800, Oberkochen, Germany). After incubating CM-H_2_DCFDA as above, the cells were fixed with 4% paraformaldehyde for 30 min and washed 3 times with PBS before photographing. The excitation and emission wavelengths were identical as described above and a minimum of five random fields was captured for each culture.

### Immunocytochemistry

Cells were washed in PBS, fixed at 4% paraformaldehyde, and permeabilized with methanol. Subsequently, they were blocked with 1% BSA for 1 h and incubated overnight with antibody against monoclonal human Rac1 at 4 °C. Alexa Fluor 488-conjugated goat antibody to mouse (Thermo Fisher Scientific) for Rac1 was used as a second antibody at 1:1000. The samples were washed, dried and mounted using Prolong Gold Antifade Reagent with DAPI (Roche, Basel, Switzerland) and visualized by Zeiss LSM 800.

### Statistical analysis

All experiments were performed in triplicate and the results were expressed as the mean ± standard deviation (SD). For comparison of the statistical differences of more than two groups, one-way ANOVA test was used and *p* values < 0.05 were considered statistically significant.

## Results

### PLAG maintains circulating neutrophils by down-regulating cell surface expression of adhesion molecules in gemcitabine-induced neutropenia model

**Fig. 1:**
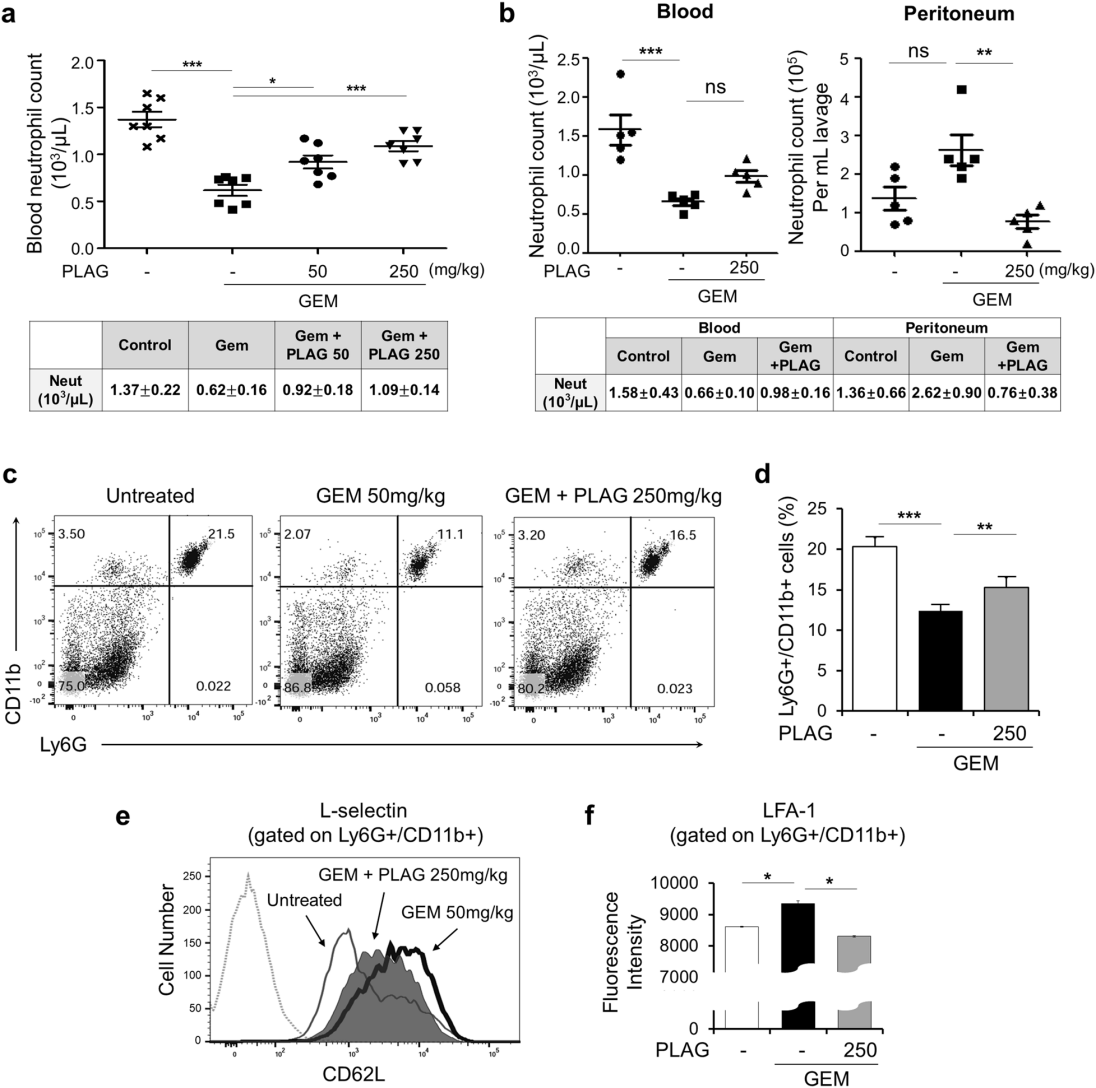
PLAG inhibits gemcitabine-induced neutrophil extravasation into the peritoneum by down-regulating the expression of adhesion molecules in normal BALB/c mice. **a** Male BALB/c mice of 6–8 weeks of age were orally administrated with 50 or 250 mg/kg of PLAG, and then were intraperitoneally injected with 50 mg/kg gemcitabine. After 24 h, blood samples were collected by retro-orbital bleeding, and the number of blood neutrophils were determined by CBC analysis. Each group contains seven mice, and bars represent the mean ± SD. **b** The number of neutrophils from the blood and the peritoneal cavity was compared. Each group contains five mice. **c**, **d** The population of blood neutrophils was analyzed by flow cytometry. Red cell-lysed whole blood was stained with FITC-conjugated anti-Ly6G and PE-Cy7-conjugated anti-CD11b antibodies to determine the circulating neutrophil population. Ly6G+/CD11b+ cells were further stained with **e** APC-conjugated anti-L-selectin and **f** APC-conjugated anti-LFA-1 antibodies and were analyzed by flow cytometry to determine the expression of adhesion molecules. *ns* not significant, *p < 0.05, **p < 0.01, ***p < 0.001

To investigate whether PLAG has an effect on gemcitabine-induced neutropenia, we orally administrated the mice with PLAG (50 and 250 mg/kg) just before gemcitabine treatment (i.p. injection; 50 mg/kg). After 15 h, gemcitabine induced a sharp decrease of circulating neutrophil counts compared to the untreated control, and administration of PLAG restored circulating neutrophils to an almost normal range in a dose dependent manner (Fig. 1a). When neutropenia was induced by other chemotherapeutic agents, 5-fluorouacil or AC regimen (adriamycin and cyclophosphamide), PLAG also effectively preserved the number of circulating neutrophils (Additional file 1: Fig. S3a and b). We also examined the number of neutrophils in the peritoneal cavity, and observed that PLAG effectively decreases neutrophil counts in the peritoneum that were elevated 15 h after gemcitabine treatment (Fig. 1b). The administration of PLAG alone did not affect the neutrophil count in either the blood or the peritoneum (Additional file 1: Fig. S2a).

Since the peritoneal neutrophil counts increased while the blood neutrophil counts decreased following gemcitabine treatment, we next investigated whether gemcitabine provokes neutrophil extravasation by up-regulating cell surface expression of adhesion molecules. First, we confirmed that PLAG maintained the population of Gr-1-positive (Gr-1^+^) and CD11b-positive (CD11b^+^) neutrophils in the blood, which was decreased by gemcitabine (Fig. 1c, d). We further examined cell surface expression of the adhesion molecules, L-selectin and LFA-1, which mediate extravasation of Gr-1^+^/CD11b^+^ cells. As a result, we found that PLAG effectively inhibited gemcitabine-induced cell surface expression of these adhesion molecules (Fig. 1e, f). These observations suggest that PLAG has a significant effect on preventing gemcitabine-induced neutrophil migration by down-regulating the surface expression of adhesion molecules.

### PLAG attenuates gemcitabine-induced expression of neutrophil-attracting chemokines MIP-2 (CXCL8) by down-regulating MAPK activation

**Fig. 2:**
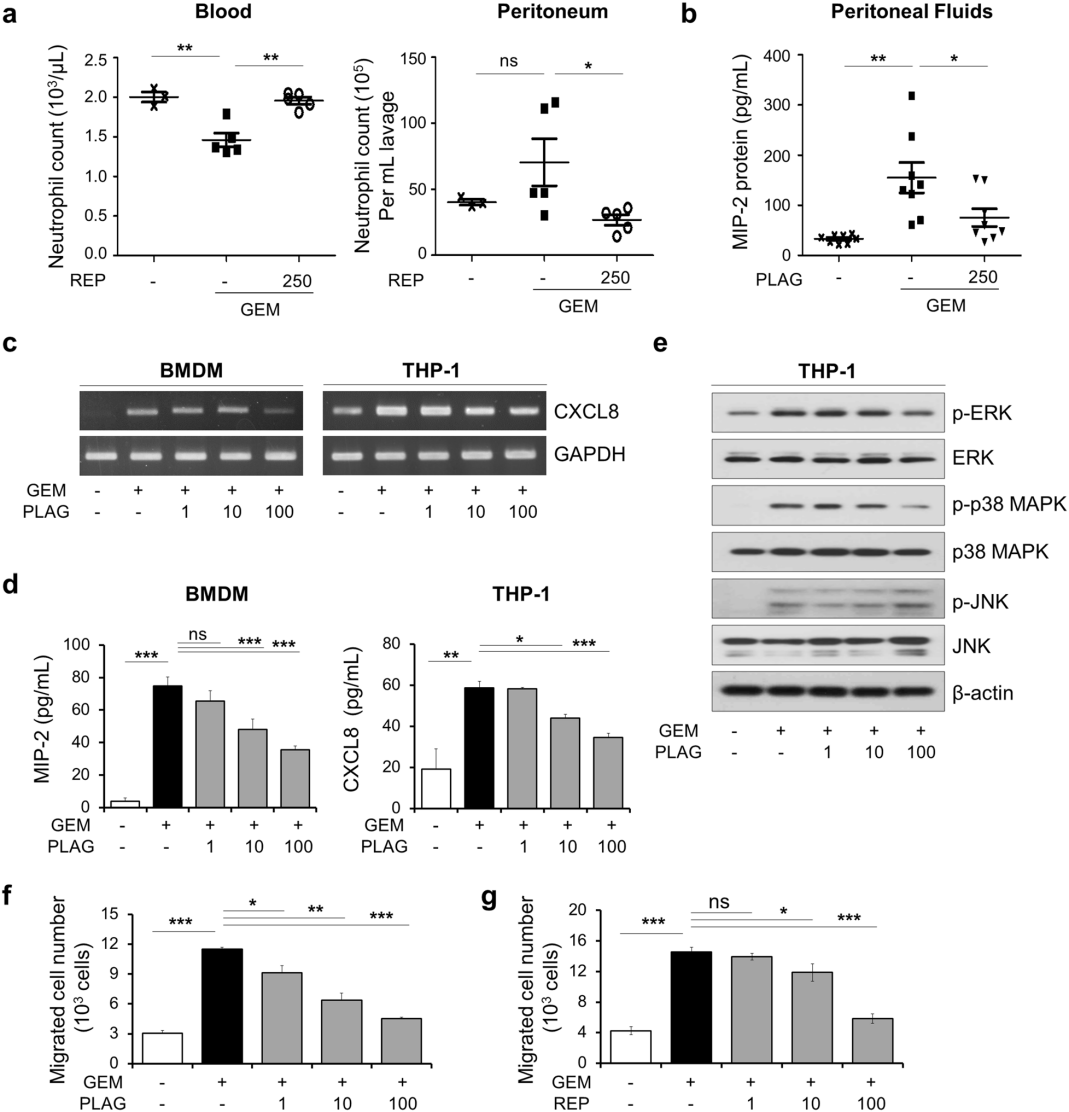
PLAG inhibits gemcitabine-induced MIP-2(CXCL8)-CXCR2-mediated neutrophil chemotaxis. **a** Reparixin was diluted in mineral oil and administered intraperitoneally at a dose of 50 mg/kg 1 h before gemcitabine administration. Neutrophils in the blood or in the peritoneal cavity were counted using CBC analysis. Each group contains five mice, and bars represent the mean ± SD. **b** Peritoneal fluids were harvested, and the protein level of MIP-2 was evaluated by ELISA. BMDMs and THP-1 cells were treated with various doses of PLAG and stimulated with gemcitabine (10 μg/ml). **c** The mRNA level of MIP-2 or CXCL8 was analyzed by RT-PCR, and **d** the protein level of MIP-2 or CXCL8 in the culture medium was determined by ELISA. **e** The effect of PLAG on gemcitabine-induced phosphorylation of ERK, p38 MAPK and JNK was analyzed by western blot in THP-1 cells. The transmigration of differentiated HL-60 cells towards THP-1 conditioned medium was decreased by **f** PLAG and **g** reparixin treatment. *ns* not significant, *p < 0.05, **p < 0.01, ***p < 0.001

Neutrophil migration is regulated by various chemokines and its receptors. The chemokine receptor CXCR2 is a major receptor responsible for neutrophil recruitment from blood to tissues during inflammation [9]. Therefore, we treated mice with reparixin, a specific noncompetitive allosteric inhibitor of CXCR2, to determine whether inactivation of this chemokine receptor on neutrophils blocks the depletion of circulating neutrophils induced by gemcitabine. Interestingly, reparixin considerably inhibited gemcitabine-induced trafficking of blood neutrophils into the peritoneal cavity (Fig. 2a). Next, we determined the protein level of mouse CXCR2 ligand, MIP-2, in the peritoneal fluids by ELISA. PLAG administration effectively reduced MIP-2 levels augmented by gemcitabine in the peritoneal fluids (Fig. 2b). To confirm our in vivo observations, we performed in vitro experiments using BMDMs and THP-1 cells. Gemcitabine increased mRNA and protein expression of CXCL8 time- and dose-dependently in THP-1 cells (Additional file 1: Fig. S3a–d). PLAG effectively inhibited gemcitabine-induced expression of MIP-2 and CXCL8 at both transcriptional and translational levels in a concentration-dependent manner (Fig. 2c, d). We next investigated the signaling pathways involved in gemcitabine-induced CXCL8 production in THP-1 cells. Gemcitabine upregulated phosphorylation of members of the mitogen-activated protein kinase (MAPK) superfamily, including ERK, p38 MAPK and JNK, in a time-dependent manner (Additional file 1: Fig. S3e). We then assessed the effect of PLAG on gemcitabine-induced phosphorylation of ERK, p38 MAPK and JNK, and observed that PLAG dose-dependently decreased the phosphorylation of ERK and p38 MAPK, but did not for JNK (Fig. 2e). Through the use of human neutrophil-like DMSO-induced differentiated HL-60 cells, we performed an in vitro cell migration assay as described in materials and methods. The conditioned medium of gemcitabine-stimulated THP-1 cells caused chemotactic migration of dHL-60 cells time- and dose-dependently (Additional file 1: Fig. S3f and g), which was inhibited by PLAG in a dose-dependent manner (Fig. 2f). When dHL-60 cells were treated with reparixin, this chemotactic activity was also diminished in a dose-dependent manner (Fig. 2g). Collectively, these observations suggest that PLAG likely attenuates neutrophil extravasation by inhibiting the gemcitabine-induced MAPK activation and chemokine production by macrophages.

### PLAG inhibits gemcitabine-induced ROS production

**Fig. 3:**
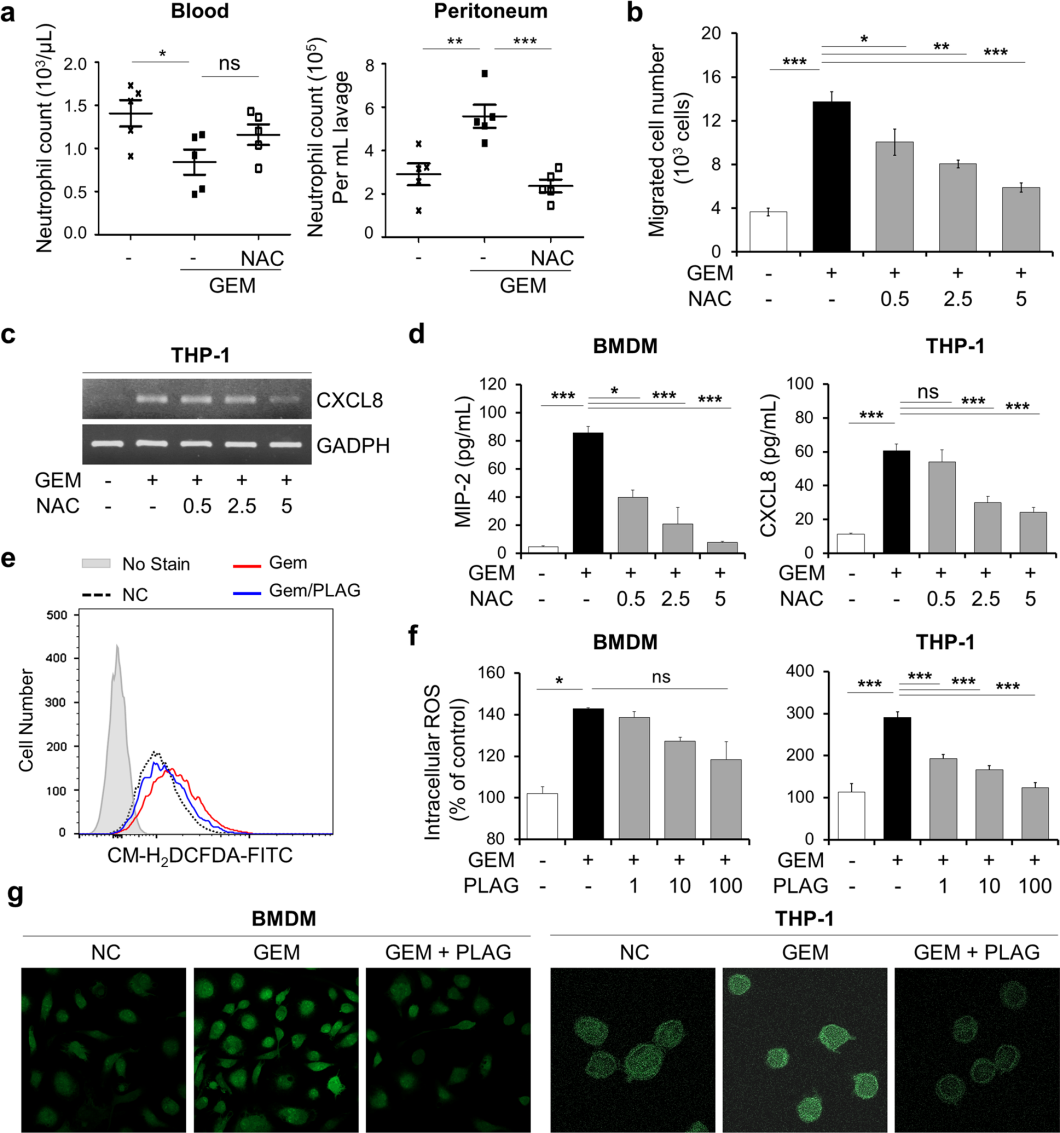
PLAG decreases gemcitabine-induced ROS production that mediates neutrophil migration. **a** NAC (50 mg/kg) was administered intraperitoneally at a dose of 50 mg/kg 1 h before gemcitabine administration. Neutrophils in the blood and the peritoneal cavity were counted using CBC analysis. Each group contains five mice, and the bars represent the mean ± SD. **b** Transmigration of dHL-60 cells towards THP-1 conditioned medium was decreased by NAC treatment. **c** The effect of NAC on gemcitabine-induced CXCL8 mRNA expression was analyzed by RT-PCR. **d** BMDMs and THP-1 cells were treated with various doses of NAC 1 h before gemcitabine treatment, and the protein level of MIP-2 or CXCL8 in the supernatants was determined by ELISA. **e**, **f** The level of intracellular ROS in BMDMs and THP-1 cells were analyzed by flow cytometry. **g** CM-H_2_DCFDA fluorescence imaging of ROS in BMDMs and THP-1 cells using a confocal microscope. *ns* not significant, *p < 0.05, **p < 0.01, ***p < 0.001

One of the mechanisms of action of gemcitabine is the generation of ROS [25]. Since it is well known that gemcitabine-generated ROS induce CXCL8 expression [26], we examined whether gemcitabine-induced ROS contributed to our neutropenia model. Interestingly, administration of a ROS scavenger, *N*-acetyl cysteine (NAC), effectively blocked gemcitabine-induced trafficking of blood neutrophils into the peritoneal cavity (Fig. 3a). An in vitro migration assay confirmed that NAC treatment to THP-1 cells decreased the chemotactic migration of dHL-60 cells in a dose-dependent fashion (Fig. 3b). In addition, NAC treatment inhibited gemcitabine-induced MIP-2 and/or CXCL8 production in BMDMs and THP-1 in a dose-dependent manner (Fig. 3c, d). By using flow cytometry and a ROS-sensitive dye, CM-H2DCFDA, which measures total cellular H_2_O_2_, we observed that gemcitabine increased intracellular ROS in a time-dependent manner (Additional file 1: Fig. S4), and PLAG effectively attenuated gemcitabine-induced intracellular ROS levels in BMDMs and THP-1 cells in a concentration-dependent manner (Fig. 3e, f). The intracellular ROS levels in BMDMs and THP-1 cells were also monitored using a confocal microscope, and we observed that PLAG attenuated gemcitabine-induced intracellular ROS production (Fig. 3g). These results indicated that gemcitabine-generated ROS is the upstream signaling molecule that induces MIP-2 and/or CXCL8 expression, and PLAG is an effective regulator for gemcitabine-induced ROS production.

### PLAG attenuates NOX2 activation by inhibiting membrane translocation of the cytosolic subunits, Rac1 and phosphorylation of p47phox

**Fig. 4:**
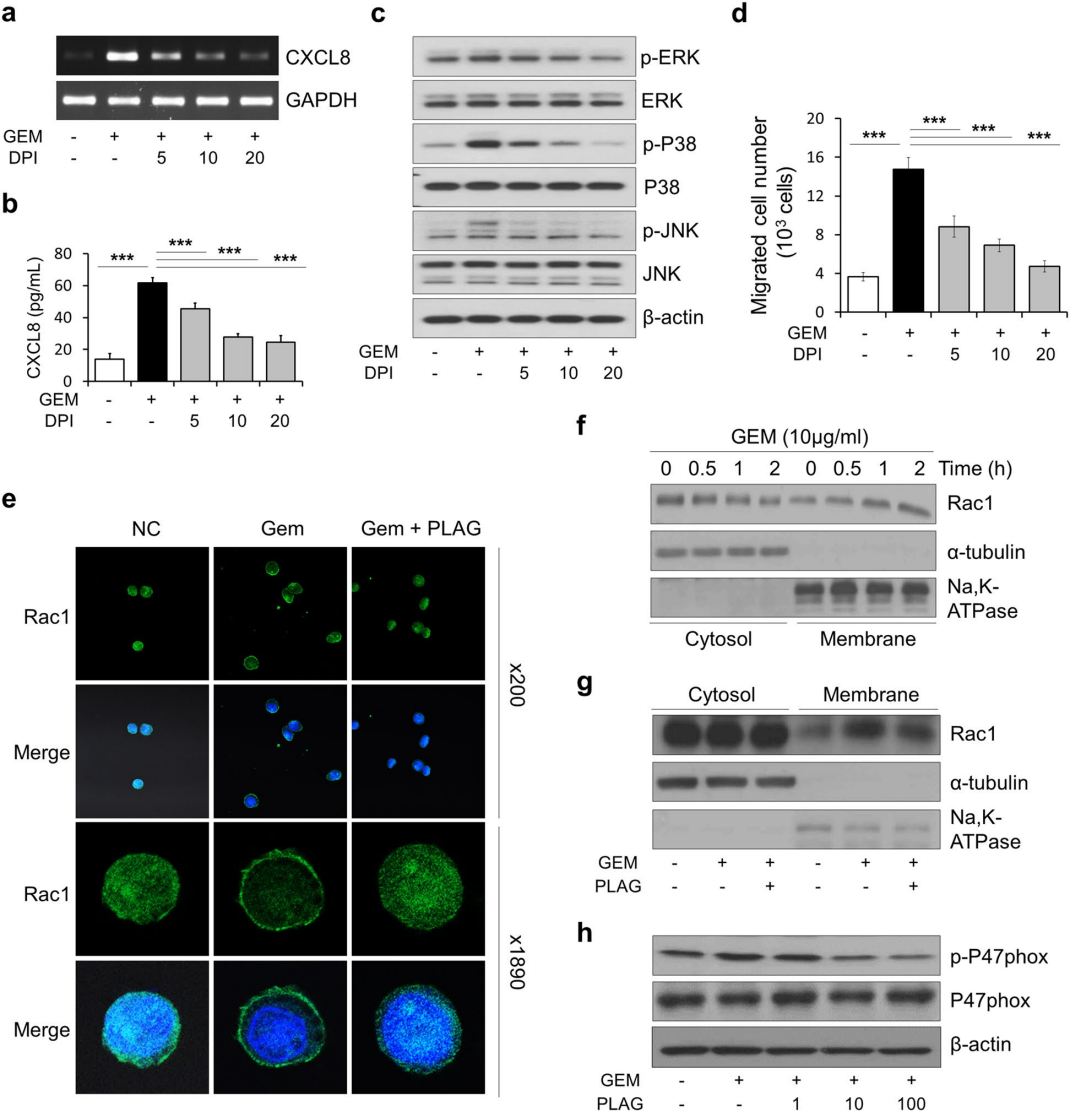
PLAG attenuates gemcitabine-induced activation of NOX2 by inhibiting membrane localization of cytosolic subunits Rac1 and p47phox phosphorylation. **a** The effect of DPI on gemcitabine-induced CXCL8 mRNA expression was analyzed by RT-PCR. **b** THP-1 cells were treated with various doses of DPI 1 h before gemcitabine treatment, and the protein level of CXCL8 in the supernatant was determined by ELISA. **c** The effect of DPI on gemcitabine-induced phosphorylation of ERK, p38 MAPK and JNK was analyzed by western blot in THP-1 cells. **d** Transmigration of dHL-60 cells towards THP-1 conditioned medium was decreased by DPI treatment. **e** THP-1 cells were treated with 100 μg/ml of PLAG 1 h before gemcitabine treatment, and then were processed for immunofluorescence with anti-Rac1 antibody. The same cells were also stained with DAPI to visualize nuclei. **f** THP-1 cells were stimulated with 10 μg/ml of gemcitabine for 0 to 2 h, or **g** the cells were pretreated with PLAG for 1 h and then stimulated with gemcitabine for 2 h, and the cytosolic and membrane proteins were fractioned as described in “Materials and methods”. The separated proteins were subject to western blot analysis and blotted with antibodies against: Rac1, Na/K-ATPase, and α-tubulin. **h** Whole lysates of THP-1 cells were separated by SDS-PAGE and subject to western blot. The blot was probed with anti-phospho-p47^phox^ antibody. *p < 0.05, **p < 0.01, ***p < 0.001

NOX2 is the main isoform expressed in both monocytes and macrophages [27], and is composed of a membrane-bound enzyme complex and several cytosolic subunits that assemble at membrane sites during the activation process to generate ROS [28]. Since a few studies reported that gemcitabine-induced ROS production is through NOX activation [17, 18, 25], we therefore assessed the involvement of NOX2 in gemcitabine-induced ROS production by treating THP-1 cells with diphenyleneiodonium (DPI), a potent and reversible NOX inhibitor. First, we measured the mRNA and protein levels of CXCL8 produced by the cells in response to gemcitabine treatment, and DPI effectively inhibited gemcitabine-induced CXCL8 production (Fig. 4a, b). DPI not only inhibited gemcitabine-induced phosphorylation of ERK and p38 MAPK, but also gemcitabine-induced JNK phosphorylation, which was not inhibited by PLAG (Fig. 4c). In vitro cell migration assay showed that treatment with DPI caused similar results as NAC treatment, suggesting that gemcitabine-induced ROS production is mediated by activation of NOX2 (Fig. 4d). The small GTPase Rac1 and p47phox are cytosolic subunits of NOX2 in phagocytes [29]. To measure translocation of these subunits from the cytosol to the membrane, we performed immunocytochemistry using confocal laser scanning microscopy. As seen in Fig. 4e, PLAG remarkably prevented gemcitabine-induced Rac1 membrane translocation in BMDMs and THP-1 cells. The membrane and cytosolic fractions isolated from gemcitabine- and/or PLAG-stimulated THP-1 cells confirmed that gemcitabine increased membrane translocation of Rac1 in a time-dependent manner (Fig. 4f), and PLAG significantly inhibited translocation of Rac1 from the cytosol to the membrane (Fig. 4g). The cytosolic component of p47phox migrates instantly to the membrane upon stimulation and assembles with the membrane components to form the active enzyme. This process is tightly regulated by the phosphorylation of p47phox. Next, we examined the effect of PLAG on gemcitabine-induced phosphorylation of p47phox, and as expected, PLAG effectively inhibited p47phox phosphorylation in a dose-dependent manner (Fig. 4h). These data indicate that PLAG decreases gemcitabine-generated ROS production by inhibiting the activation of NOX2 via inhibition of Rac1 membrane translocation and p47phox phosphorylation.

### PLAG inhibits gemcitabine-induced phosphorylation of PLCβ3, PKCα/βII and PKCδ, which are upstream signaling regulators of p47phox phosphorylation

**Fig. 5:**
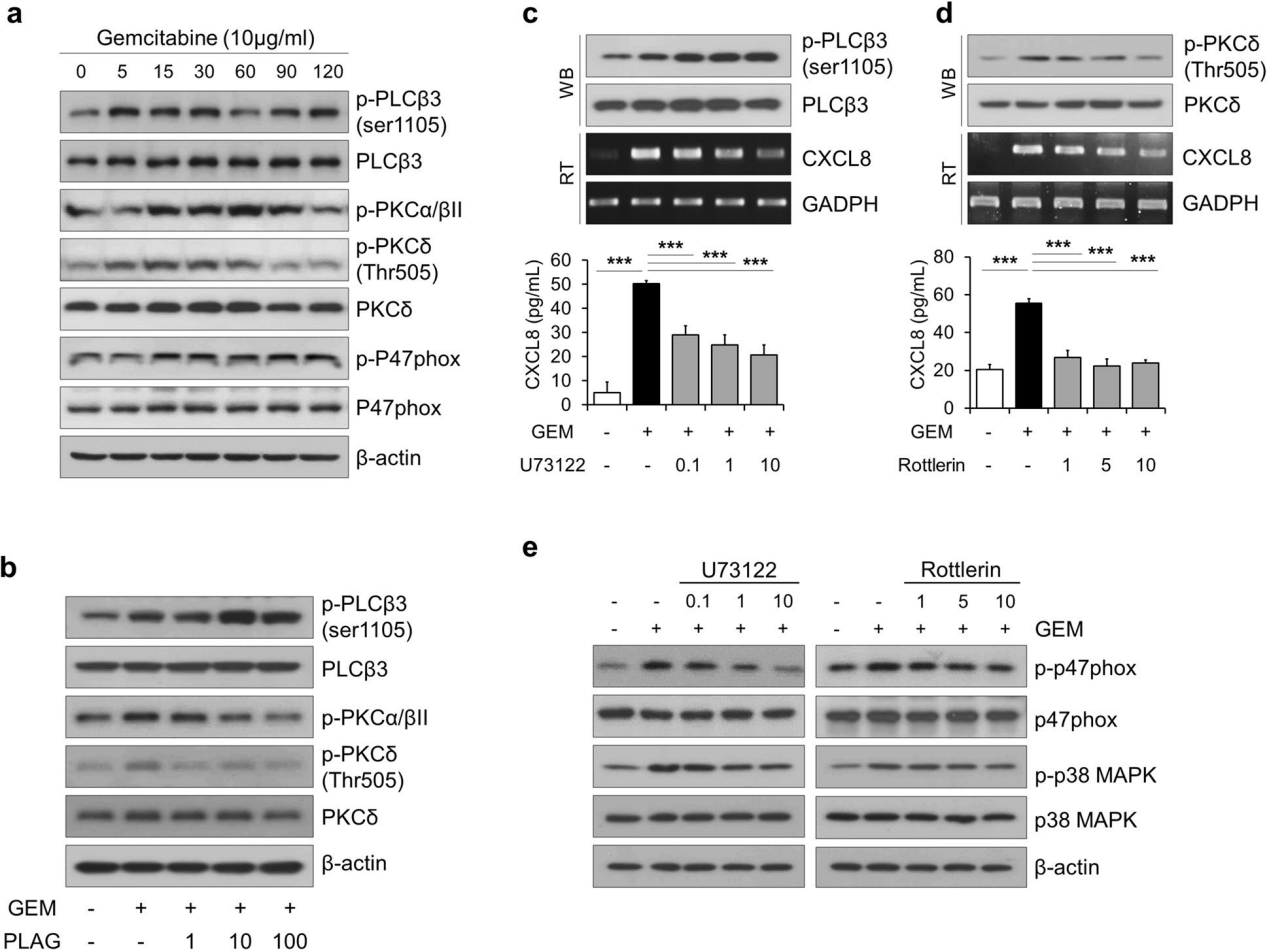
PLAG inhibits gemcitabine-induced phosphorylation of PLCβ3, PKCα/βII and PKCδ, which are upstream signaling regulators of p47phox phosphorylation. **a** THP-1 cells were treated with 10 μg/ml of gemcitabine for 0 to 2 h, and the phosphorylation of PLCβ3, PKCα/βII, PKCδ and p47phox was analyzed by western blot. **b** The effect of PLAG on gemcitabine-induced phosphorylation of PLCβ3, PKCα/βII and PKCδ was analyzed by western blot. THP-1 cells were treated with **c** PLC inhibitor (U73122) and **d** PKC inhibitor (Rottlerin) 1 h before gemcitabine treatment, and the mRNA and the protein level of CXCL8 and **e** p47phox and p38 MAPK phosphorylation were determined. *p < 0.05, **p < 0.01, ***p < 0.001

We next investigated the effect of PLAG in the upstream signaling pathways that modulate NOX2 activation. Since several isoforms of protein kinase C (PKC) are required for p47phox phosphorylation and translocation, we examined whether gemcitabine induces the phosphorylation of PKC alpha/beta II and PKC delta. We observed that gemcitabine treatment for 0 to 120 min induced the phosphorylation of PKC alpha/beta II at Thr638/641 and the phosphorylation of PKC delta at Thr505 (Fig. 5a). Most PKC family molecules are activated by calcium and/or diacylglycerol (DAG), which are the by-products of hydrolysis of phospholipids by phospholipase C (PLC). Gemcitabine also increased the phosphorylation of PLC beta 3 at ser1105 (Fig. 5a), while PLC gamma was not phosphorylated by gemcitabine (data not shown). PLAG dose-dependently increased the phosphorylation of PLC beta 3 at serine1105, indicating that PLAG effectively inhibited the activity of PLC beta 3 (Fig. 5b). PLAG also down-regulated gemcitabine-induced phosphorylation of PKC alpha/beta II and PKC delta in a dose-dependent manner (Fig. 5b). To confirm the signal transduction pathways that regulate NOX2 activation and CXCL8 production, we pretreated THP-1 cells with a PLC inhibitor U73122 and a PKC inhibitor Rottlerin 1 h before gemcitabine treatment. Treatment with these chemicals dose-dependently inhibited gemcitabine-induced transcriptional and translational CXCL8 production (Fig. 5c, d). In addition, these chemicals effectively inhibited gemcitabine-induced phosphorylation of p47phox and p38MAPK (Fig. 5e). These results indicated that PLAG effectively regulated gemcitabine-induced activation of upstream signaling regulators, PLC beta 3, PKC alpha/beta II and PKC delta, of p47phox phosphorylation.

### The acetylated form of PLAG stimulates dHL-60 transmigration

**Fig. 6:**
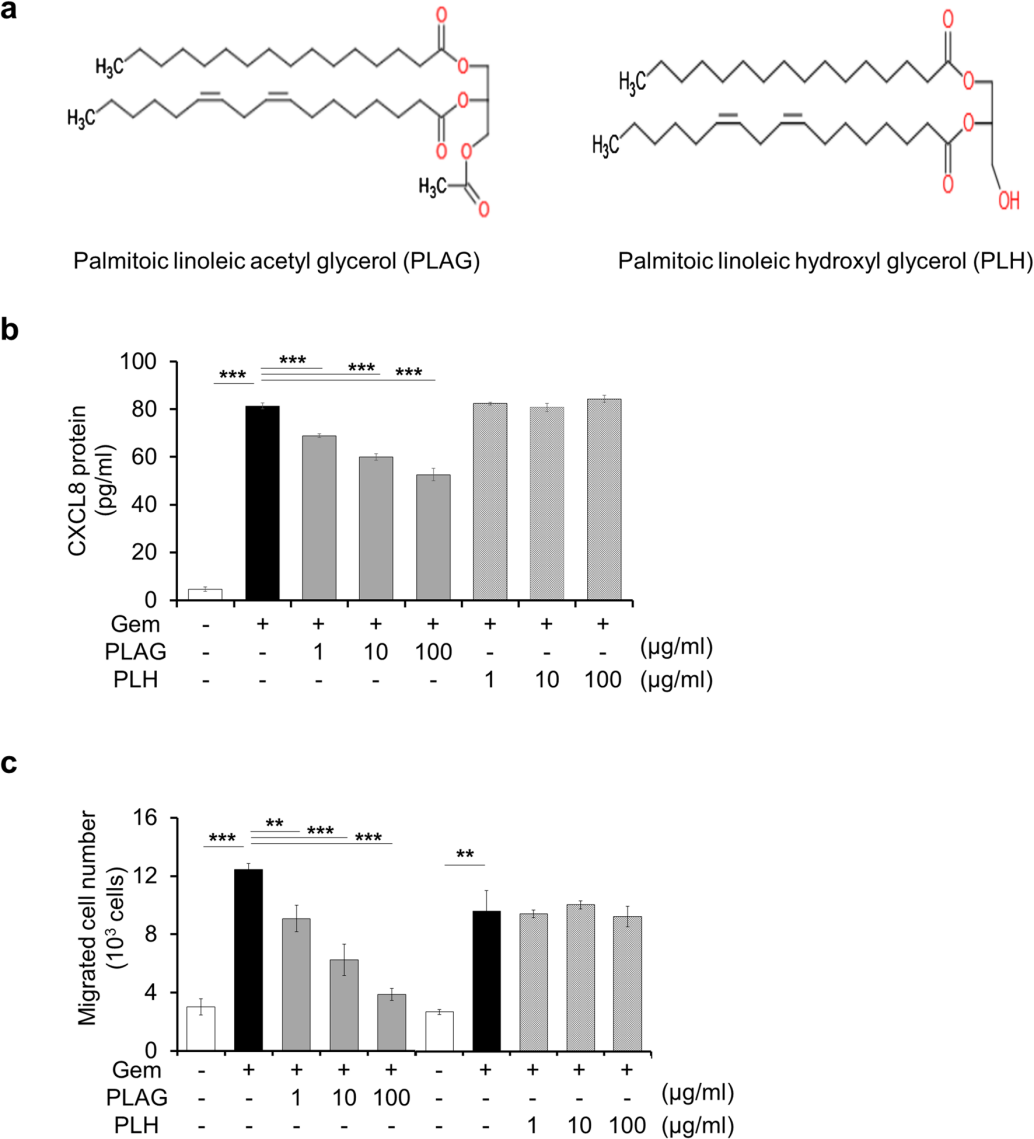
Selectivity of PLAG in the inhibition of gemcitabine-induced CXCL8 production and dHL-60 cell migration in vitro. **a** Chemical structures of PLAG and PLH. **b** Gemcitabine-induced CXCL8 production was decreased by PLAG, but not PLH. **c** Transmigration of dHL-60 cells was decreased by PLAG, but not PLH. *p < 0.05, **p < 0.01, ***p < 0.001

PLAG is an 1,2-acyl-3-acetylglycerol-type lipid molecule. Its structure is different from diacylglycerol and triacylglycerol in that the third position of glycerol backbone is esterified with acetyl group. Next, we investigated whether the effects of PLAG result from the acetyl group by comparing with its un-acetylated form, palmitoic linoleic hydroxyl glycerol (PLH). Figure 6a shows the chemical structure of PLAG and PLH. THP-1 cells were pretreated with 1, 10, and 100 μg/ml of PLH or PLAG for 1 h then stimulated with gemcitabine for 24 h. We first evaluated the effects of PLAG and PLH on the level of CXCL8 secretion. As a result, whereas PLAG inhibited gemcitabine-induced CXCL8 secretion dose-dependently, PLH had no effects on it (Fig. 6b). For migration assay, the conditioned medium from THP-1 cells was placed on the bottom of the wells, dHL-60 cells were added to the upper chamber, and migration was measured. While the migration of dHL-60 cells was inhibited by PLAG in a dose-dependent manner, PLH had no inhibitory effects on dHL-60 cell migration (Fig. 6c). Based on these observations, we suggest that the acetyl group in PLAG may play a key role in the inhibition of gemcitabine-induced CXCL8 production and dHL-60 migration.

### PLAG does not interfere with the anti-cancer effect of gemcitabine

It is well known that chemotherapeutic agents often elevate the intracellular level of ROS [30], which plays a doubled-edged sword role in both normal and cancer cells [17]. Gemcitabine-induced ROS is also an important mechanism of action for tumor killing [31]. For this reason, there is a concern whether the inhibition effect of PLAG on ROS generation might reduce the anti-tumor effect of gemcitabine. Therefore, we investigated how PLAG affects tumor growth when combined with gemcitabine treatment in athymic nude mice implanted with a human multiple myeloma cell line, RPMI8226 cells. Additional file 1: Fig. S6a shows a schematic diagram of the experiment. As a result, we observed that PLAG 500 mg/kg-treated group exhibited significantly less tumor growth compared to the negative control group at day 40. Also, gemcitabine 80 or 120 mg/kg-treated group showed significantly less tumor growth compared to the negative control group. Addition of PLAG to gemcitabine, however, did not exhibit differences in tumor weight compared to gemcitabine only treatment (Additional file 1: Fig. 6b). This result indicated that the administration PLAG does not interfere with the tumor killing effect of gemcitabine.

## Discussion

Gemcitabine is a chemotherapeutic agent used widely for various malignant tumor types including pancreatic cancer [15]. During chemotherapy, genotoxic agent—like gemcitabine—generally induce neutropenia, which occurs when circulating neutrophil number falls below the absolute neutrophil count in the blood, necessitating dose reduction or cessation of chemotherapy [32]. Neutropenic patients are more vulnerable to infection because circulating neutrophils play a key role in the first-line of defense against invading pathogens [3]. In spite of its importance, the mechanism for CIN has not been well elucidated. In this study, we demonstrate that extravasation of circulating neutrophils is promoted by chemokine gradients produced by gemcitabine treatment, which may be one of the major causes of CIN. Importantly, we also show that PLAG blocks excessive neutrophil egress and maintains circulating neutrophils in the blood, making it a potential therapeutic option for patients with CIN.

Several studies demonstrated that gemcitabine-induced ROS acted as an upstream signaling molecule in the activation of mitogen-activated protein kinase (MAPK) signaling pathways [33–35]. We also observed that gemcitabine induced the phosphorylation of ERK1/2, P38, and JNK, which is effectively inhibited by PLAG and a NADPH inhibitor, DPI. One discrepancy between PLAG and DPI is that gemcitabine-induced JNK phosphorylation is inhibited by DPI but not by PLAG. While PLAG specifically inhibits NOX2-mediated ROS production, DPI is a potent NOX inhibitor that inactivates flavoenzymes of all NOX isoforms [36]. In addition, DPI is known to potently reduce mitochondrial ROS production through inhibiting NADH-ubiquinone oxidoreductase [37]. We presume that other sources of ROS besides NOX2 contribute to gemcitabine-induced JNK activation.

Excessive ROS production by neutrophils causes the vascular endothelium to open inter-endothelial junctions and to promote the extravasation of inflammatory cells across the endothelial vessels [38]. In addition, ROS, especially hydrogen peroxide (H_2_O_2_), not only relay signals from the immune cell surface to activate adhesion molecules for cell movement, but also act in non-migrating cells to influence the behavior of migrating cells [39]. We demonstrated the effect of PLAG on down-regulation of adhesion molecules of neutrophils mainly attributed to the reduced amount of MIP-2 and/or CXCL8 chemokines produced by resident macrophages. However, we acknowledge that this would not be the only mechanism regulating the migration of neutrophils. Therefore, it is necessary to study how other different types of cells, such as endothelial cells, respond to chemotherapeutic agents and influence neutrophil migration and activation.

PLAG is a synthetic form of 1,2-acyl-3-acetylglycerol, which naturally occurs in trace amounts in a broad range of organisms, including the antlers of sika deer (*Cervus Nippon*), the udders of domestic cows (*Bos primigenius*), and the seeds of the burning bush plant (*Euonymus alatus*) [40–42]. The third position in the glycerol backbone of PLAG is esterified with an acetyl group, which may confer distinct features on this form compared to the three-chain fatty acid esterified triacylglycerol or diacylglycerol with two-chain fatty acid esterified. Many studies have demonstrated the therapeutic efficacy of PLAG in various disease models. Our previous study showed that PLAG exerts a synergistic effect with pegfilgrastim to treat CIN without promoting neutrophil release from bone marrow or inducing surface expression of CXCR2, implying that the mechanism of PLAG is different from that of pegfilgrastim [22]. We also identified that PLAG has an anti-inflammatory effect in an OVA-induced allergic asthma model by attenuating CCL26 expression from lung epithelial cells and eosinophil infiltration into the respiratory tract [24]. In a 5-fluorouracil-induced oral mucositis model, PLAG enhanced the formation of newly differentiated epidermis and blood vessels in the cheek pouch by decreasing infiltration of inflammatory cells and mucositis-associated weight loss [43]. Taken together, PLAG has a remarkable effect on regulating excessive immune activation in a variety of inflammation-associated diseases.

Here, we show that CIN is the consequence of excessive circulating neutrophil transmigration in response to chemotactic gradients produced by resident macrophages, and that PLAG has remarkable effects on CIN prevention through the regulation of PLCβ3/PKC/NOX2/ROS/MAPK signaling cascades. Therefore, we expect that PLAG could be a new potential therapeutic strategy for CIN.

## Additional file

**Additional file 1.** **Figure S1.** Analysis of Differentiation of BMDM and human neutrophil-like HL-60 cells by flow cytometry. **A,** FACS analysis illustrates the purity of BMDM at day 7 using a macrophage marker F4/80-PE. **B,** Differentiation of HL-60 cells into human neutrophil-like cells confirmed by flow cytometry and a CD11b-PE marker. **Figure S2.** Neutrophil counts of the blood and peritoneum in mice treated with PLAG, reparixin or NAC. Male balb/c mice of 6 to 8 weeks of age were orally administered with 250mg/kg of PLAG (in PBS), or intraperitoneally injected with 50mg/kg of reparixin (in mineral oil) or with 50mg/kg of NAC (in PBS). After 15h, blood and peritoneal fluid samples were collected for CBC analysis. The number of neutrophils from the blood and the peritoneal fluid of **(A)** PLAG, **(B)** reparixin and **(C)** NAC-treated mice. Each group contains five mice, and bars represent the mean ± SD. ns, not significant. **Figure S3.** PLAG inhibits other chemotherapeutic agents-induced neutrophil migration. Male balb/c mice of 6-8 weeks of age were orally administrated with 250mg/kg PLAG, and then intraperitoneally injected with **(A)** 100mg/kg 5-fluouracil or **(B)** AC regimen (2.5mg/kg of adriamycin and 100mg/kg of cyclophosphamide) for 24h. The number of blood neutrophils were determined by CBC analysis. Each group contains five mice, and bars represent the mean ± SD. * p<0.05, ** p<0.01, *** p<0.001. **Figure S4.** Gemcitabine induces a neutrophil-attracting chemokine CXCL8 production via MAPK activation in THP-1 cells. The mRNA level of CXCL8 in human monocytic THP-1 stimulated with **(A)** various doses and **(B)** different time points of gemcitabine treatment. The protein concentration of CXCL8 in THP-1 stimulated with **(C)** various doses and **(D)** different time points of gemcitabine treatment. **E,** Gemcitabine induces phosphorylation of ERK, p38 MAPK and JNK analyzed by western blot in THP-1 cells. The transmigration of differentiated HL-60 cells towards the conditioned medium of THP-1 stimulated with **(F)** various doses and **(G)** different time points of gemcitabine treatment. * *p*<0.05, ** *p*<0.01, *** *p*<0.001. **Figure S5.** Gemcitabine increases intracellular reactive oxygen species (ROS) levels in a time-dependent manner. **A and B,** THP-1 cells were treated with 10ug/ml of gemcitabine for different time points, and then the level of intracellular ROS in the cells was stained with CM-H2DCFDA and analyzed by flow cytometry. **C,** CM-H_2_DCFDA fluorescence imaging of ROS in THP-1 cells using a confocal microscope. The bars represent the mean ± SD. * p<0.05, ** p<0.01, *** p<0.001. **Figure S6.** PLAG does not interfere with the anti-cancer effect of gemcitabine in athymic nude mice implanted with a human myeloma cell line RPMI8226. **A,** The treatment design and schedule using a human RPMI8226 xenograft mice model. **B,** The tumor weights of the treatment groups at day 40. ns; not significant, *p < 0.05.
