# Supplementary material for: 1-Palmitoyl-2-linoleoyl-3-acetyl-rac-glycerol (PLAG) attenuates gemcitabine-induced neutrophil extravasation
Source: Cell Biosci. 2019 Jan 3;9:4. doi: 10.1186/s13578-018-0266-7 (PMC6317242; doi:10.1186/s13578-018-0266-7)
Supplement: Supplementary file 1 — Additional file 1. Figure S1. Analysis of Differentiation of BMDM and human neutrophil-like HL-60 cells by flow cytometry. A, FACS analysis illustrates the purity of BMDM at day 7 using a macrophage marker F4/80-PE. B, Differentiation of HL-60 cells into human neutrophil-like cells confirmed by flow cytometry and a CD11b-PE marker. Figure S2. Neutrophil counts of the blood and peritoneum in mice treated with PLAG, reparixin or NAC. Male balb/c mice of 6 to 8 weeks of age were orally administered with 250mg/kg of PLAG (in PBS), or intraperitoneally injected with 50mg/kg of reparixin (in mineral oil) or with 50mg/kg of NAC (in PBS). After 15h, blood and peritoneal fluid samples were collected for CBC analysis. The number of neutrophils from the blood and the peritoneal fluid of (A) PLAG, (B) reparixin and (C) NAC-treated mice. Each group contains five mice, and bars represent the mean ± SD. ns, not significant. Figure S3. PLAG inhibits other chemotherapeutic agents-induced neutrophil migration. Male balb/c mice of 6-8 weeks of age were orally administrated with 250mg/kg PLAG, and then intraperitoneally injected with (A) 100mg/kg 5-fluouracil or (B) AC regimen (2.5mg/kg of adriamycin and 100mg/kg of cyclophosphamide) for 24h. The number of blood neutrophils were determined by CBC analysis. Each group contains five mice, and bars represent the mean ± SD. * p<0.05, ** p<0.01, *** p<0.001. Figure S4. Gemcitabine induces a neutrophil-attracting chemokine CXCL8 production via MAPK activation in THP-1 cells. The mRNA level of CXCL8 in human monocytic THP-1 stimulated with (A) various doses and (B) different time points of gemcitabine treatment. The protein concentration of CXCL8 in THP-1 stimulated with (C) various doses and (D) different time points of gemcitabine treatment. E, Gemcitabine induces phosphorylation of ERK, p38 MAPK and JNK analyzed by western blot in THP-1 cells. The transmigration of differentiated HL-60 cells towards the conditioned medium of THP-1 stimulat [file 13578_2018_266_MOESM1_ESM.pptx]

## Slide 1
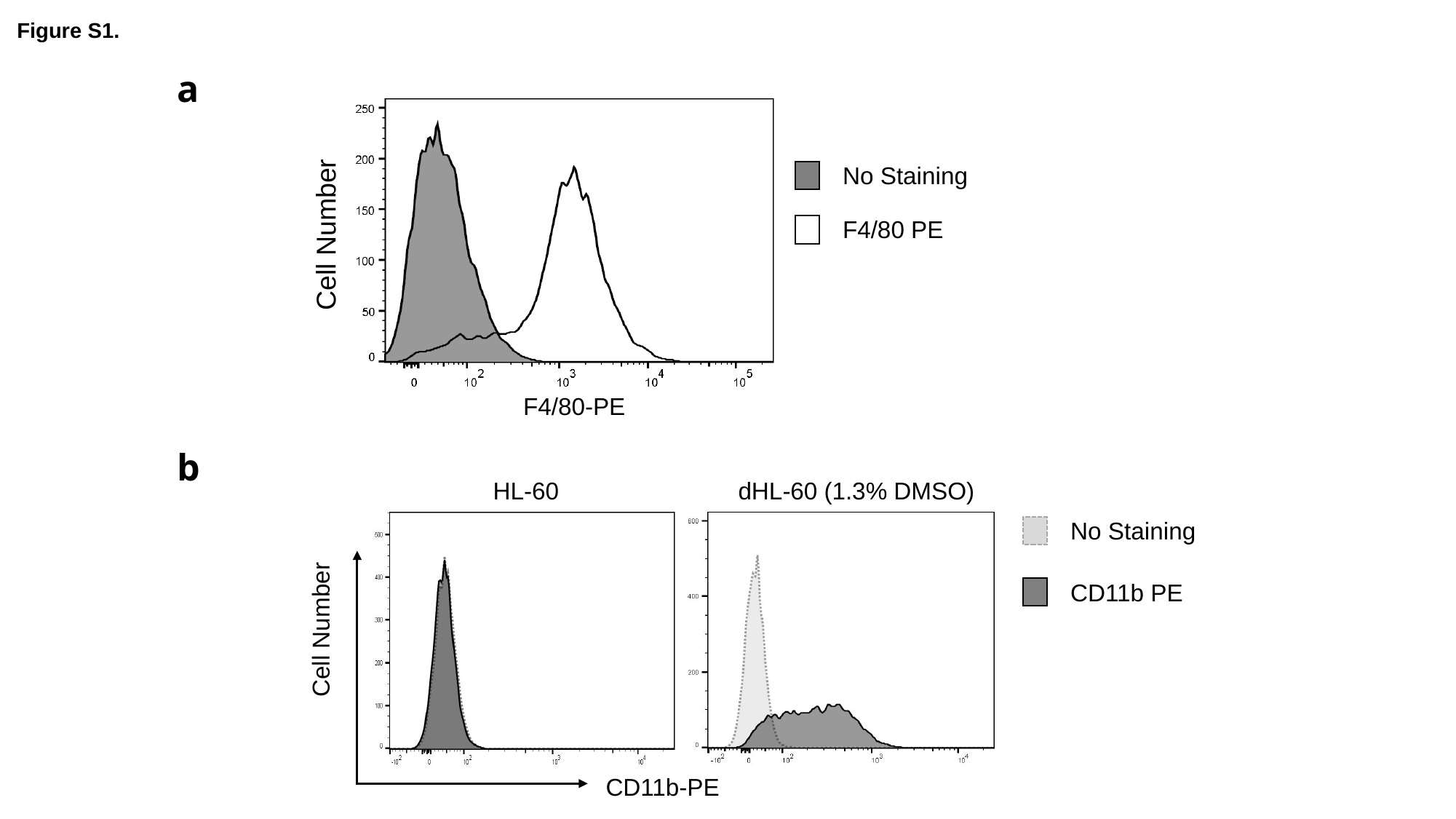

Figure S1.
a
No Staining
F4/80 PE
Cell Number
F4/80-PE
b
HL-60
dHL-60 (1.3% DMSO)
No Staining
CD11b PE
Cell Number
CD11b-PE

## Slide 2
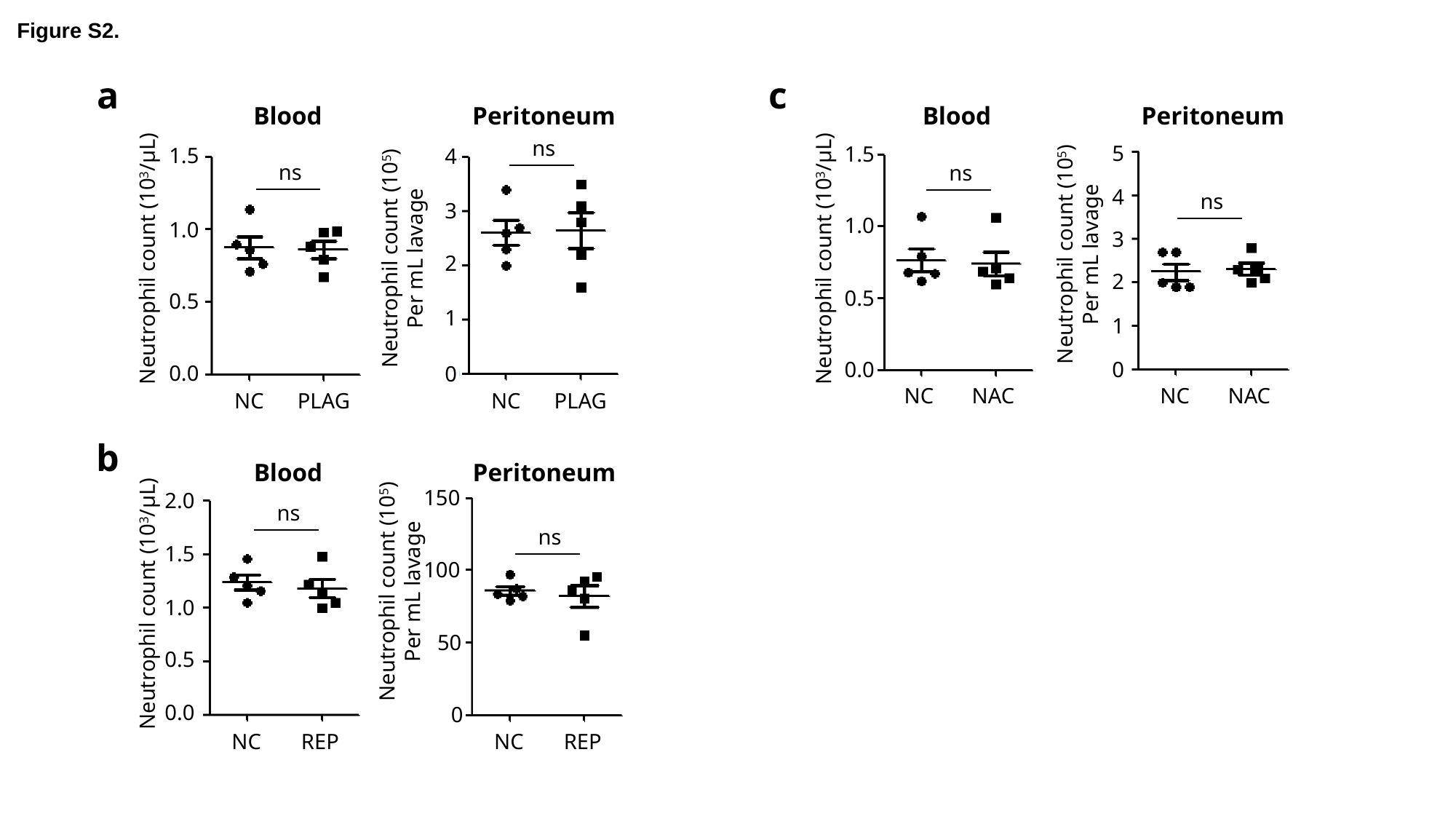

Figure S2.
a
c
Blood
Peritoneum
Blood
Peritoneum
1.5
4
3
1.0
Neutrophil count (105)
Per mL lavage
Neutrophil count (103/μL)
2
0.5
1
0.0
0
5
1.5
4
1.0
3
Neutrophil count (105)
Per mL lavage
Neutrophil count (103/μL)
2
0.5
1
0
0.0
ns
ns
ns
ns
NC
NAC
NC
NAC
NC
PLAG
NC
PLAG
b
Blood
Peritoneum
150
2.0
1.5
100
Neutrophil count (105)
Per mL lavage
Neutrophil count (103/μL)
1.0
50
0.5
0.0
0
ns
ns
NC
REP
NC
REP

## Slide 3
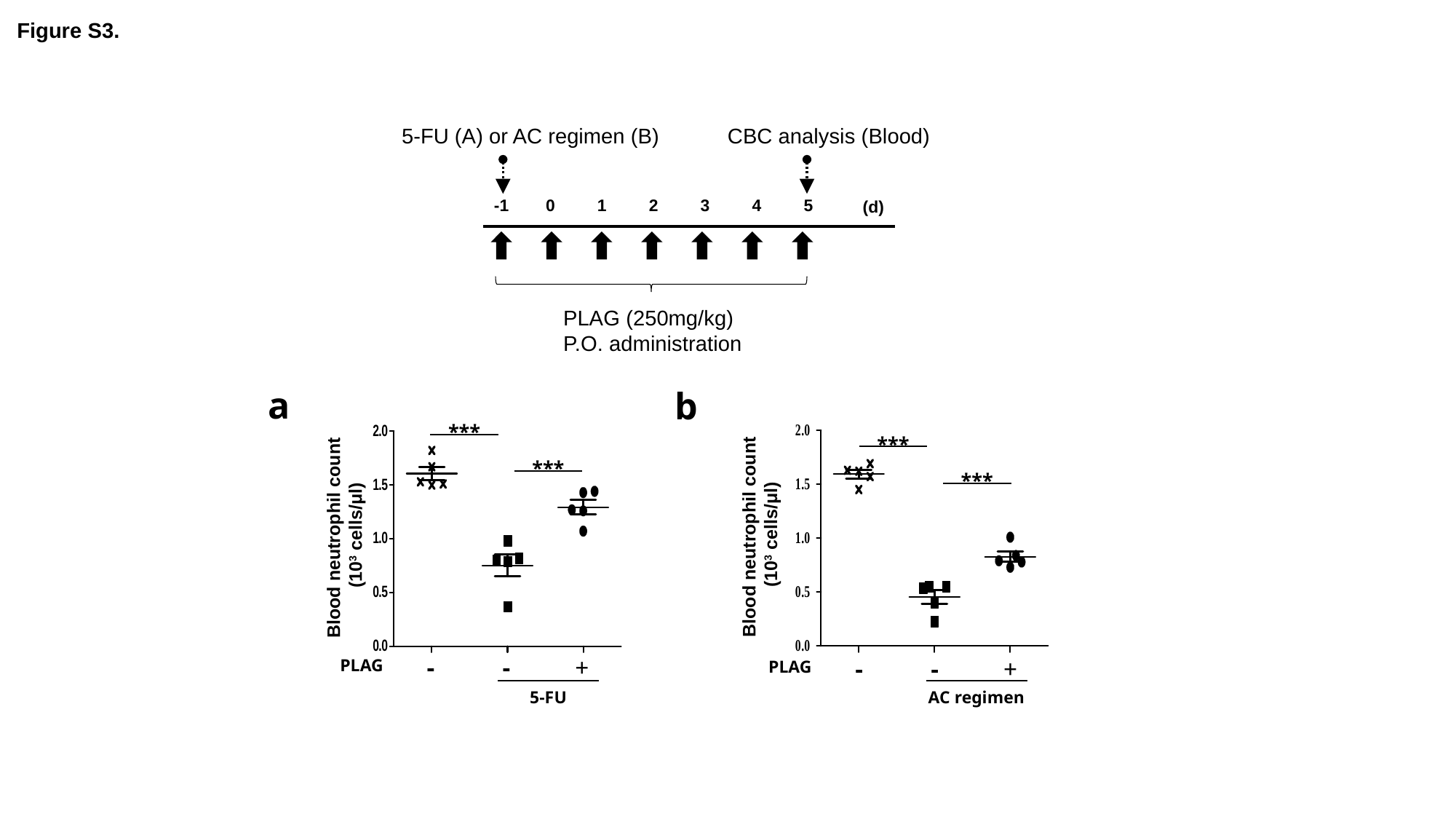

Figure S3.
5-FU (A) or AC regimen (B)
CBC analysis (Blood)
| -1 | 0 | 1 | 2 | 3 | 4 | 5 |
| --- | --- | --- | --- | --- | --- | --- |
(d)
PLAG (250mg/kg)
P.O. administration
a
b
***
***
***
***
Blood neutrophil count
(103 cells/μl)
Blood neutrophil count
(103 cells/μl)
| PLAG | - | - | + |
| --- | --- | --- | --- |
| PLAG | - | - | + |
| --- | --- | --- | --- |
5-FU
AC regimen

## Slide 4
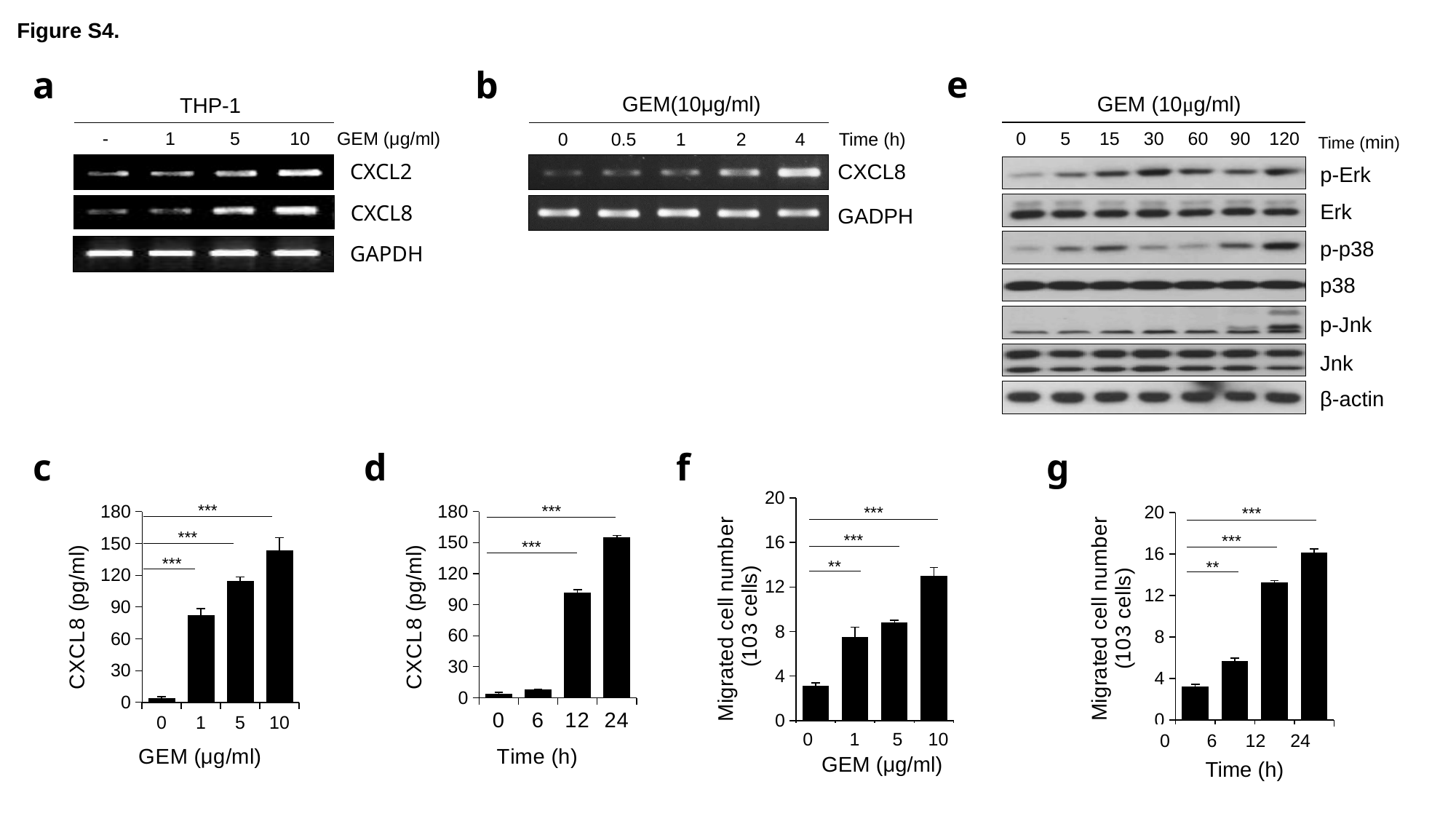

Figure S4.
e
a
b
GEM(10μg/ml)
GEM (10μg/ml)
THP-1
| - | 1 | 5 | 10 | GEM (μg/ml) |
| --- | --- | --- | --- | --- |
0
0.5
1
2
4
Time (h)
| 0 | 5 | 15 | 30 | 60 | 90 | 120 |
| --- | --- | --- | --- | --- | --- | --- |
Time (min)
CXCL8
CXCL2
p-Erk
Erk
CXCL8
GADPH
p-p38
GAPDH
p38
p-Jnk
Jnk
β-actin
c
d
f
g
### Chart
| Category | |
|---|---|
### Chart
| Category | |
|---|---|***
***
### Chart
| Category | |
|---|---|
| 0 | 3.3840579710144922 |
| 6 | 7.949275362318838 |
| 12 | 101.39130434782611 |
| 24 | 154.54347826086953 |
### Chart
| Category | |
|---|---|
| 0 | 3.813333333333333 |
| 1 | 81.67999999999999 |
| 5 | 114.21333333333331 |
| 10 | 142.7466666666666 |***
***
***
***
***
***
***
**
**
| 0 | 1 | 5 | 10 |
| --- | --- | --- | --- |
| 0 | 6 | 12 | 24 |
| --- | --- | --- | --- |
GEM (μg/ml)
Time (h)

## Slide 5
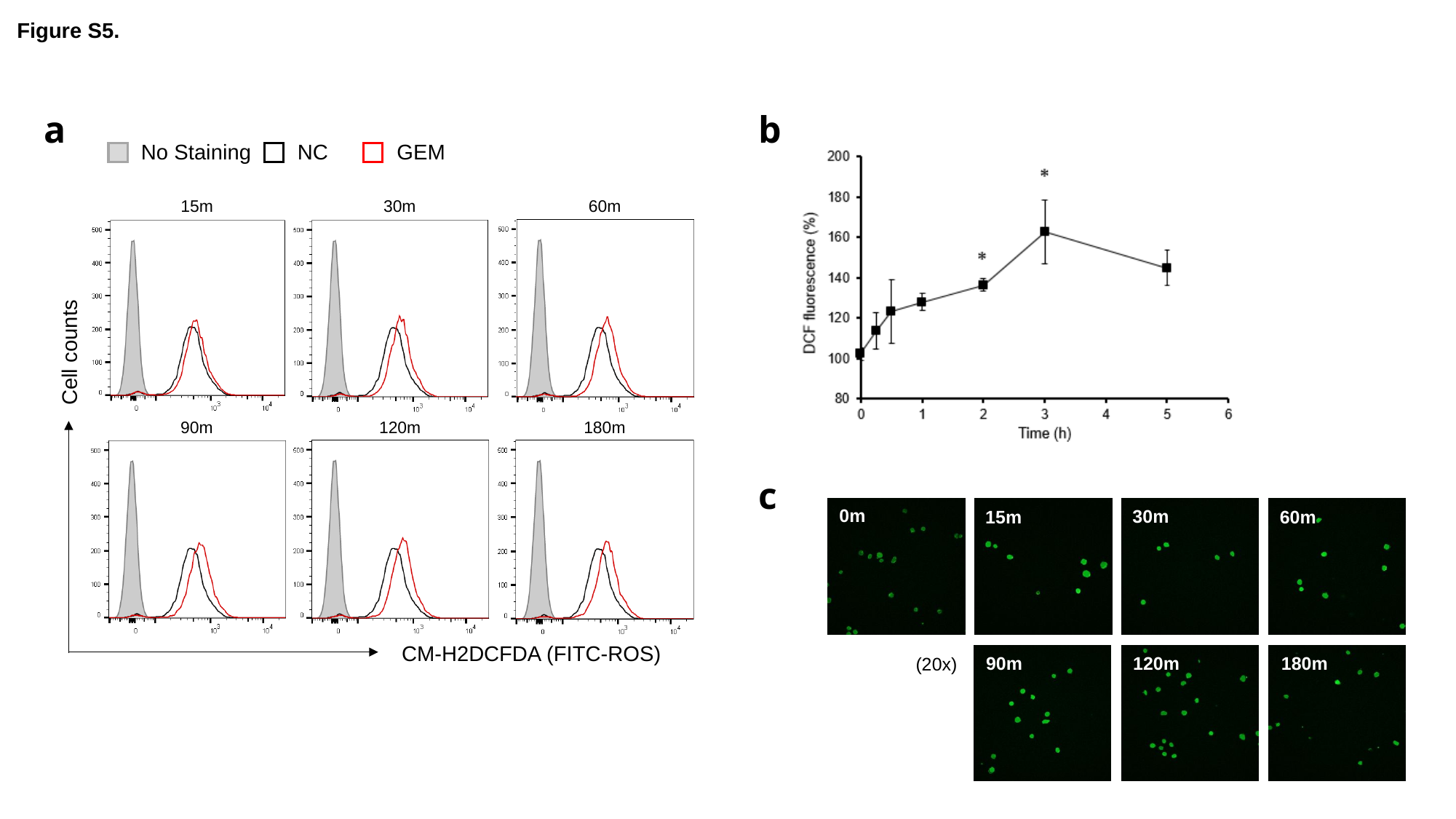

Figure S5.
a
b
NC
GEM
No Staining
15m
30m
60m
Cell counts
90m
120m
180m
CM-H2DCFDA (FITC-ROS)
c
0m
30m
15m
60m
90m
180m
120m
(20x)

## Slide 6
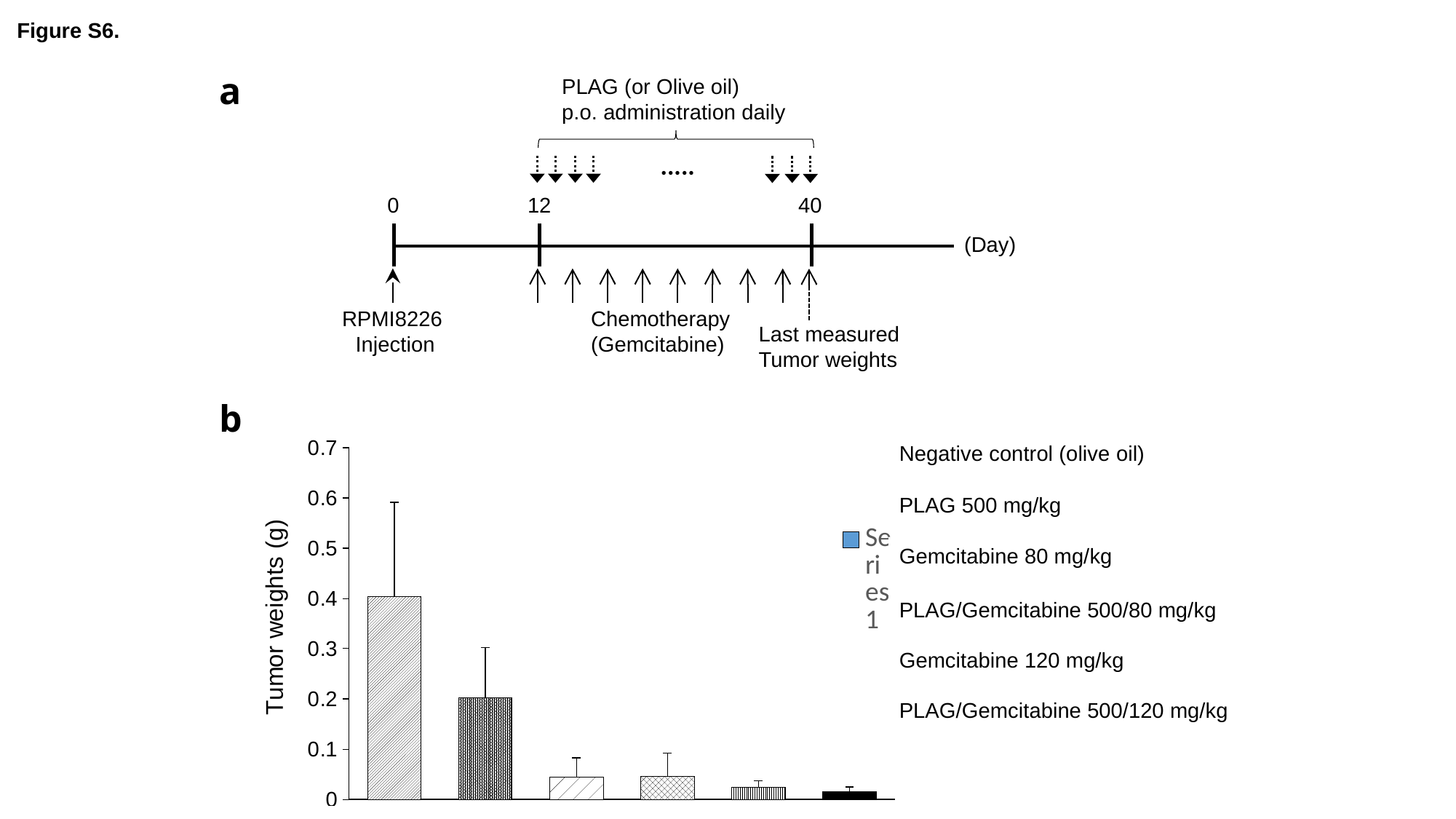

Figure S6.
a
PLAG (or Olive oil)
p.o. administration daily
·····
0
12
40
(Day)
RPMI8226
Injection
Chemotherapy
(Gemcitabine)
Last measured
Tumor weights
b
### Chart
| Category | |
|---|---|
Negative control (olive oil)
PLAG 500 mg/kg
Gemcitabine 80 mg/kg
PLAG/Gemcitabine 500/80 mg/kg
Gemcitabine 120 mg/kg
PLAG/Gemcitabine 500/120 mg/kg
